# Nevus Lipomatosus Cutaneous Superficialis Mimicking an Acrochordon

**DOI:** 10.7759/cureus.13554

**Published:** 2021-02-25

**Authors:** Lennie To, Thomas Vazquez, Nicole Izhakoff, Martin Zaiac

**Affiliations:** 1 Department of Dermatology, Florida International University, Herbert Wertheim College of Medicine, Miami, USA; 2 Dermatology, Greater Miami Skin and Laser Center, Miami Beach, USA

**Keywords:** nevus lipomatosus superficialis, nevus, acrochordon, hamartoma, adipocyte, shave biopsy, nlcs

## Abstract

Nevus lipomatosus cutaneous superficialis (NLCS) is a rare cutaneous hamartoma characterized by mature adipocytes in the dermis. Here, we present a unique case of NLCS in a 57-year-old female that strikingly resembled an acrochordon, demonstrating features of the classical and solitary form of NLCS. This presentation of NLCS has not been widely reported and emphasizes that the diagnosis of NLCS should be considered when evaluating suspected acrochordons.

## Introduction

Nevus lipomatosus cutaneous superficialis (NLCS) is a rare cutaneous hamartoma characterized by mature adipocytes in the papillary or reticular dermis [1,2]. Clinically, there are two forms of NLCS. The classical form is more common and consists of multiple soft, elastic, skin-colored, cerebriform, and pedunculated papules or nodules that can coalesce to form plaques with surfaces that may be wrinkled, smooth, or have a peau d’orange appearance [2]. The classical form usually appears in the pelvic and gluteal regions, but there have been several reported cases on the face [2,3]. The rarer solitary form is characterized as a sessile, dome-shaped nodule or papule that appears in variable locations, but has a predilection for the trunk [2,3]. This form of NLCS most often appears after the second decade of life [3]. Lesions of NLCS typically do not change in size, but may progress if untreated [1].

NLCS is almost always asymptomatic and has not been associated with malignant changes; however, there are reports of NLCS causing ulnar nerve entrapment as well as ulcerating after external trauma. NLCS has also been associated with multiple cutaneous disorders such as follicular papules, hypertrophic pilosebaceous units, angiokeratoma of Fordyce, café-au-lait macules, scattered leukoderma, and hemangioma [1].

The histopathology of NLCS is characterized by the presence of ectopic fat in the reticular and papillary dermis, making up 10-50% of the lesion [4]. This usually does not extend to the subcutaneous adipose tissue [1]. The adipocytes usually form aggregates around merocrine glands or blood vessels but may be present between collagen bundles as well [4]. Increased pigmentation, acanthosis, and basket weave hyperkeratosis have been seen in the epidermis, but changes to the epidermis are not always present [1].

## Case presentation

**Figure 1:**
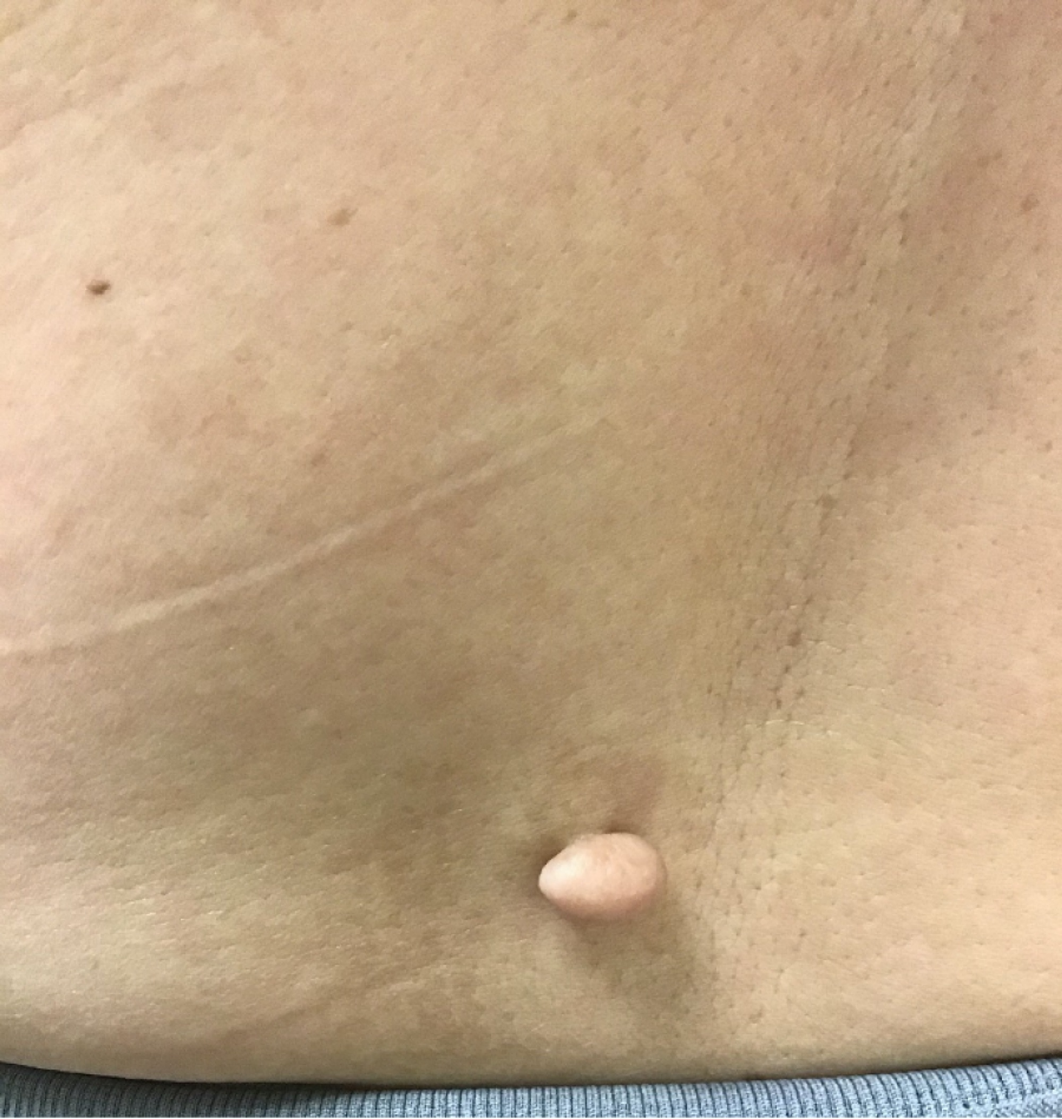
NLCS on the sacral area that closely resembles an acrochordon. NLCS, nevus lipomatosus cutaneous superficialis

A 57-year-old female presented with a non-painful mass on her lower back which she stated was present for some time. Physical examination revealed a solitary skin-colored, pedunculated nodule with a smooth surface on the sacral area (Figure 1). The nodule was somewhat rubbery. A shave biopsy was performed and a diagnosis of an NLCS was made.

We report a case of a solitary NLCS that strikingly resembled an acrochordon, including the fibrolipomatous variant [5]. A diagnosis of NLCS initially seemed unlikely given its appearance. The solitary form is commonly sessile and dome-shaped, whereas the pedunculated lesions associated with the classical form are typically cerebriform (in contrast to our patient’s lesion which was smooth).

## Discussion

The pathogenesis of NLCS is still unclear. Hoffman and Zurhelle postulated that the deposition of adipocytes in the dermis is secondary to degenerative alterations in connective tissue [2]. Other theories suggest ectopic adipocyte origination from the pericytes of dermal vessels, adipose metaplasia in the connective tissue within the dermis, or developmental displacement of adipose tissue [1-4]. The genetic background of NLCS has not been clearly described, but one study has shown a cytogenic aberration associated with a 2p24 deletion [1].

We believe this case is unique as it highlights features of both the classical and solitary form of NLCS, placing it within the wider differential diagnosis for skin-colored pedunculated lesions (Table 1) [2,3]. A speedy clinical diagnosis of acrochordon would have left the patient with an incorrect diagnosis, leading to a potential for patient apprehension, whereas acrochordons are well known to be associated with insulin resistance and obesity, even having the potential to be a marker for metabolic syndrome [1]. When evaluating suspected acrochordons, we believe this case demonstrates that a diagnosis of NLCS should be entertained.

**Table 1:** Lesions that may resemble NLCS. NLCS, nevus lipomatosus cutaneous superficialis

Differential diagnoses for NLCS
Connective nevus
Nevus sebaceous
Verrucous nevus
Plexiform neurofibroma
Smooth muscle hamartoma
Leiomyoma cutis
Neurofibroma
Lipomatosis
Lipoblastomatosis
Acrochordon

## Conclusions

NLCS is managed with surgical excision. However, if left untreated, NLCS can increase in size, causing apprehension. Unlike acrochordons, NLCS is not associated with insulin resistance or metabolic syndrome. Patients with NLCS should be reassured that this condition is benign and not a known marker for other underlying conditions. In addition, further research is needed to properly classify NLCS subtypes and recognize that certain lesions may not fit well into existing classifications. Dermatologists and other practitioners should be aware of the possibility of an NLCS diagnosis when evaluating a patient with an isolated, pedunculated skin-colored nodule which may have the appearance of an acrochordon. Atypical acrochordae should be sent for histological review when a diagnosis of NLCS is possible.
